# Development of a Novel ATP Bioluminescence Assay Based on Engineered Probiotic *Saccharomyces boulardii* Expressing Firefly Luciferase

**DOI:** 10.4014/jmb.2305.05019

**Published:** 2023-07-27

**Authors:** Ji Sun Park, Young-Woo Kim, Hyungdong Kim, Sun-Ki Kim, Kyeongsoon Park

**Affiliations:** 1 Department of Systems Biotechnology, Chung-Ang University, Anseong 17546, Republic of Korea; 2 Department of Food Science and Technology, Chung-Ang University, Anseong 17546, Republic of Korea

**Keywords:** *Saccharomyces boulardii*, whole-cell biocatalyst, ATP detection, inflammatory bowel disease

## Abstract

Quantitative analysis of adenosine triphosphate (ATP) has been widely used as a diagnostic tool in the food and medical industries. Particularly, the pathogenesis of a few diseases including inflammatory bowel disease (IBD) is closely related to high ATP concentrations. A bioluminescent D-luciferin/luciferase system, which includes a luciferase (FLuc) from the firefly *Photinus pyralis* as a key component, is the most commonly used method for the detection and quantification of ATP. Here, instead of isolating FLuc produced in recombinant *Escherichia coli*, we aimed to develop a whole-cell biocatalyst system that does not require extraction and purification of FLuc. To this end, the gene coding for FLuc was introduced into the genome of probiotic *Saccharomyces boulardii* using the CRISPR/Cas9-based genome editing system. The linear relationship (r^2^ = 0.9561) between ATP levels and bioluminescence generated from the engineered *S. boulardii* expressing FLuc was observed in vitro. To explore the feasibility of using the engineered *S. boulardii* expressing FLuc as a whole-cell biosensor to detect inflammation biomarker (*i.e.*, ATP) in the gut, a colitis mouse model was established using dextran sodium sulfate as a colitogenic compound. Our findings demonstrated that the whole-cell biosensor can detect elevated ATP levels during gut inflammation in mice. Therefore, the simple and powerful method developed herein could be applied for non-invasive IBD diagnosis.

## Introduction

Adenosine triphosphate (ATP) is the main source of energy in all living cells and is therefore a crucial mediator of various biological processes [1]. Given that the viability and metabolic activity of cells and the presence of factors affecting cellular metabolism are closely related to ATP concentration, ATP-metry methods have been widely used in the food, biotechnology, and diagnostic industries [2]. Furthermore, ATP plays important roles in the pathogenesis of a variety of human diseases, such as arterial high blood pressure diseases and inflammatory bowel disease (IBD), through purinergic signaling [3, 4]. Therefore, the determination of ATP concentration can provide key insights into the pathogenesis of ATP-related diseases.

**Fig. 1:**
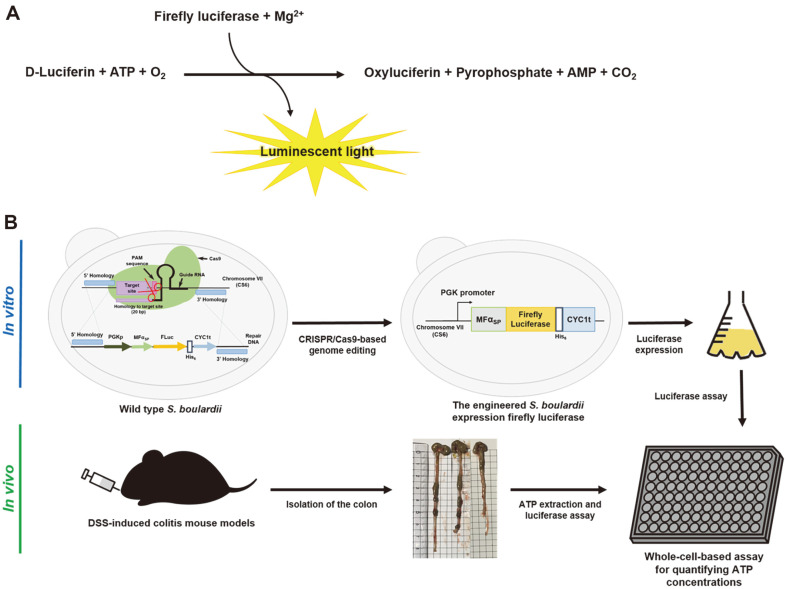
Bioluminescent reaction catalyzed by firefly luciferase (A) and schematic representation of the overall procedure for in vitro and in vivo assays (B). ATP, adenosine triphosphate; AMP, adenosine monophosphate; MFα_SP_, mating factor alpha signal peptide; DSS, dextran sulfate sodium.

Among the various methods for determining ATP concentration, a bioluminescent D-luciferin/luciferase system is the most widely used method due to its high sensitivity and specificity [5]. The luciferase derived from the firefly *Photinus pyralis* catalyzes the oxygenation of D-luciferin to oxyluciferin in the presence of ATP, producing yellow–green light with a peak at 560 nm (Fig. 1A). The firefly luciferase (FLuc) used in the ATP assay is currently produced in recombinant *Escherichia coli* strains [6, 7]. However, this production system requires extraction and purification of FLuc, which increases production costs. Therefore, we sought to develop a whole-cell biocatalysis system in which engineered *Saccharomyces cerevisiae* var. *boulardii* cells expressing FLuc are directly used for the detection of ATP in the presence of D-luciferin.

*S. cerevisiae* and *S. boulardii* share more than 99% of genome sequence homology [8] and these yeast strains have been widely used as eukaryotic hosts to produce various proteins [9, 10]. Among these two yeast strains, however, only *S. boulardii* displays probiotic activity and has been used for the treatment of gastrointestinal tract disorders [11]. In this study, we introduced a FLuc expression cassette into the genome of *S. boulardii* using the clustered regularly interspaced short palindromic repeat (CRISPR)/Cas9-based genome editing system. Then, the applicability of the engineered *S. boulardii* expressing FLuc was examined both in vitro and in vivo (Fig. 1B). Because the ATP concentration is closely related to the development of IBD [3, 4], a dextran sulfate sodium (DSS)-induced colitis mouse model was used to conduct in vivo experiments. Additionally, given that *S. boulardii* is eliminated from the gut within 2–5 days after discontinuing its administration [12], the engineered *S. boulardii* expressing FLuc has great potential as an *in situ* imaging-based diagnostic tool for IBD diagnosis and monitoring.

## Materials and Methods

### Strains and Plasmids

The *Escherichia coli* TOP10 strain (Invitrogen, USA) was used for gene cloning. *S. boulardii* ATCC MYA-796 was used for FLuc expression. The *S. boulardii* strains and plasmids used in this study are listed in Table 1.

### Chemicals and Materials

D-luciferin potassium salt was purchased from GoldBio (USA). ATP disodium salt was obtained from Daejung (Korea). Dextran sulfate sodium salt (DSS; molecular weight = 40,000) was obtained from Alfa Aesar (USA). Penicillin-streptomycin solution and gentamicin were purchased from Welgene Inc. (Korea) and Gibco (USA), respectively. Phosphate buffered saline (PBS) and 10% formaldehyde were acquired from Lonza (Basel, Switzerland) and Sigma-Aldrich (USA), respectively.

### Genetic Manipulation

All cloning experiments were performed using the NEBuilder HiFi DNA Assembly Master Mix (New England Biolabs, USA) as specified by the manufacturer. The pFLuc plasmid containing the FLuc expression cassette was constructed in three cloning steps. First, a DNA fragment coding for the mating factor alpha (MFα) signal peptide was amplified via polymerase chain reaction (PCR) with the JC01 and JC03 primers using pMFα_SP_-UreA as a template. This DNA fragment was combined with a linearized pTPO1 plasmid [13] containing a *GPD* promoter (GPDp) and a *CYC1* terminator (CYC1t) amplified with JC02 and JC04 primers to construct the pJCK01 intermediate plasmid. Second, to replace GPDp with the *PGK* promoter (PGKp) in the pJCK01 plasmid, a DNA fragment without GPDp was amplified with JC01 and JC06 primers using the pJCK01 plasmid as a template. This DNA fragment was combined with a DNA fragment coding for PGKp, which was amplified with the JC05 and JC07 primers using *S. cerevisiae* genomic DNA as a template. These two linear DNA fragments were combined using NEBuilder HiFi DNA Assembly Master Mix to construct the pJCK02 intermediate plasmid. In the last step, a DNA fragment containing the gene coding for FLuc was amplified via PCR with the KH15 and KH16 primers using pTH652-RLuc/maxFLuc (Addgene plasmid #29698) as a template. This DNA fragment was combined with a linearized pJCK02 plasmid amplified with the KH13 and KH14 primers to construct the pFLuc plasmid.

To introduce the FLuc expression cassette (PGKp-MFα_SP_-FLuc-His_6_-CYC1t) into the CS6 locus [14], an intergenic region between *HIP1* and the gene coding for tRNA in chromosome VII of *S. boulardii*, the CRISPR/Cas9-based genome editing system was used as previously described [15]. Briefly, to construct a guide RNA plasmid, a DNA fragment containing the guide RNA expression cassette was amplified with the SK37 and SK38 primers using pgRNA-*TRP1*-HYB [16] as a template. The resulting PCR product was combined using NEBuilder HiFi DNA Assembly Master Mix to construct the pgRNA-CS6-HYB plasmid. Additionally, a repair DNA fragment containing the FLuc expression cassette was amplified with the SK35 and SK36 primers using the pFLuc plasmid as a template. The resulting repair DNA fragment and the pgRNA-CS6-HYB plasmid were co-transformed into *S. boulardii* ATCC MYA-796 harboring pCas9_AUR [17] to construct the SKSC175 strain (Table 1). The successful insertion of the FLuc expression cassette into the *S. boulardii* chromosome was verified using PCR amplification with the SK33 and SK34 primers, followed by sequencing of the PCR products. All primers used for genetic manipulation are listed in Table S1.

### Media and Culture Conditions

*E. coli* cells were cultivated at 37°C and 230 rpm in Luria-Bertani (LB) medium (5 g/l yeast extract, 10 g/l tryptone, and 10 g/l NaCl) containing 50 μg/ml ampicillin. *S. boulardii* strains were pre-cultured at 30°C and 230 rpm for 48 h in yeast peptone (YP) medium (10 g/l of yeast extract and 20 g/l of Bacto Peptone) containing 20 g/l glucose. The pre-cultured cells were harvested and inoculated into main cultures with an initial optical density at 600 nm (OD_600_) of 1.0. The main fermentation experiments were conducted at 30°C and 230 rpm for 72 h in a baffled flask containing 100 ml of YP medium with 50 g/l glucose.

### Protein Preparation and Analysis of FLuc Expression

After cell cultivation, the culture broth was centrifuged at 12,000 rpm for 10 min to collect the extracellular protein fraction. The cells were harvested at OD_600_*ml = 50 (*i.e.*, if the OD_600_ was 10, then 5.0 ml of culture was harvested) to collect the intracellular proteins. The harvested cells were resuspended in Y-PER (Thermo Fisher Scientific, USA) and lysed according to the manufacturer’s instructions. The total and soluble protein fractions were prepared as described in a previous study [18].

To visualize the proteins, the protein samples fractionated by 12% (w/v) sodium dodecyl sulfate-polyacrylamide gel electrophoresis (SDS-PAGE) were transferred to polyvinylidene difluoride membranes (Millipore, USA). For the immunoblot assays, His_6_-fusion proteins were detected using an anti-polyhistidine antibody (Abcam, ab18184) according to the manufacturer’s instructions.

### In Vitro Detection of ATP

To evaluate whether the SKSC175 strain expressing FLuc can be used for the development of a whole-cell-based ATP detection assay, the culture broth containing the SKSC175 strain [6.64 × 10^7^ colony forming units (CFU)/ml, 50 μl] were transferred to each well of a 96-well black plate with various concentrations of ATP (1, 5, 10, 25 and 10 μM; 100 μl). To initiate the reaction, D-luciferin solution (6 mg/ml, 50 μl) was added into each well. After mild shaking, the bioluminescence intensities were measured with an integration time of 1 sec under luminescence mode using a Synergy H1 microplate reader (BioTek, USA). To prepare cell lysates, the culture broth containing the SKSC175 strain [6.64 × 10^7^ colony forming units (CFU)/ml] were suspended in PBS (pH 7.4) and then lysed by glass beads (I.D. 0.5 mm, Biospec, USA) as specified by the manufacturer. The in vitro detection of ATP using cell lysates of the SKSC175 strain was carried out as described above.

### DSS-Induced Colitis Mouse Model

All in vivo mouse experiments described herein were approved by the Institutional animal Care and Use Committee of Chung-Ang University (A2022060). An acute DSS-induced colitis mouse model was established with Balb/c mice (5-week-old, female, DooYeol Biotech, Korea) as described in previous studies with minor modifications [19, 20]. For the exposures, the mice received 3% DSS in their drinking water for 3 or 7 days. The control mice were given water only. Symptoms of colitis were assessed daily by measuring body weight, evaluating stool consistency, and detecting bloody stools. Disease severity was scored using a clinical disease activity index (DAI) ranging from 0 to 4, which was calculated as described previously [21] using the following parameters: weight loss, stool consistency, and presence or absence of fecal blood. The mice were sacrificed on day 8, and the entire colon was excised. Colon lengths in all groups were measured, after which 0.5 cm of the distal part of the colon was used for histological assessment. For histological analysis, the distal section of the colon was fixed in 10% formaldehyde, embedded into paraffin blocks, and stained with hematoxylin and eosin (H&E). The histological sections were graded based on a scoring system reported in previous studies [22, 23].

### Measurement of Extracellular ATP from Colon Tissues of DSS-Induced Colitis Model Mice Using the SKSC175 Strain Expressing FLuc

The capability of ATP detection in the colon tissues from DSS-induced colitis mice was further examined. The extracellular ATP levels in the colon tissues from colitis mice were measured as described in a previous report with minor modifications [22]. As detailed in Section 2.7, the excised colon tissues from proximal to distal sections were weighted and then kept in PBS containing penicillin (100 U/ml), streptomycin (100 μg/ml), and gentamycin (10 μg/ml) for 5 min. The tissues were then washed three times with PBS and further incubated in 10 volumes (w/v) of PBS at 37°C for 1 h, after which the supernatants were collected. The ATP levels in the collected supernatants were determined using the SKSC175 strain and D-luciferin. The SKSC175 strain (3.32 × 10^6^ CFU) suspended in 50 μl of PBS was transferred into 96-well black plates. Then, 100 μl of the collected supernatants or the ATP solution (0–200 μM) for the standard curve was added into each well, followed by 50 μl of D-luciferin solution (6 mg/ml in DW). After mild shaking, the bioluminescence intensity of each well was recorded. Finally, the ATP levels of the collected supernatants from each group were determined based on the ATP standard curve [Y = 2.882x + 18.05 (R^2^=0.9662)].

### Statistical Analysis

Statistical analyses were performed using the Systat Software (USA). All data are presented as mean ± standard deviation. Pair-wise comparisons were conducted via Student’s *t*-test. *P*-values less than 0.05 were considered statistically significant.

## Results and Discussion

### Construction of the Engineered *S. boulardii* Expressing FLuc

The SKSC175 strain containing the FLuc expression cassette in its genome was constructed using the CRISPR/Cas9-mediated allele replacement method (Fig. S1A). Extracellular secretion of FLuc was initially attempted to increase substrate accessibility by attaching the MFα signal peptide (*i.e.*, a representative signal peptide used in yeast) to the N-terminal end of FLuc (Fig. S1A). However, FLuc expression was not detected in the extracellular fraction of the SKSC175 strain (data not shown). The results of this study and earlier studies suggested that the attachment of the signal peptide alone does not guarantee extracellular secretion of target proteins [24, 25]. Nevertheless, FLuc was successfully expressed in the intracellular fraction of the SKSC175 strain in which the soluble expression level of FLuc was low compared to its total expression level, including both soluble and insoluble expression levels (Fig. S1B). This result indicates that FLuc is mainly present in an insoluble form, suggesting that this protein is insoluble and/or unstable in *S. boulardii*. Thus, additional studies are actively being conducted to enhance the soluble expression level of FLuc in *S. boulardii*. Chaperone co-expression [26], signal peptide optimization [27], and fusion protein technology [28] can be used to improve solubility and secretion efficiency of FLuc. Given the presence of membrane transporters for ATP uptake in yeast [29] and the cell-permeable property of D-luciferin, the SKSC175 strains could be used as a critical component for the whole-cell-based ATP detection assay, in which the intensity of the bioluminescence was directly proportional to the ATP concentration.

### In Vitro Detection of ATP

**Fig. 2:**
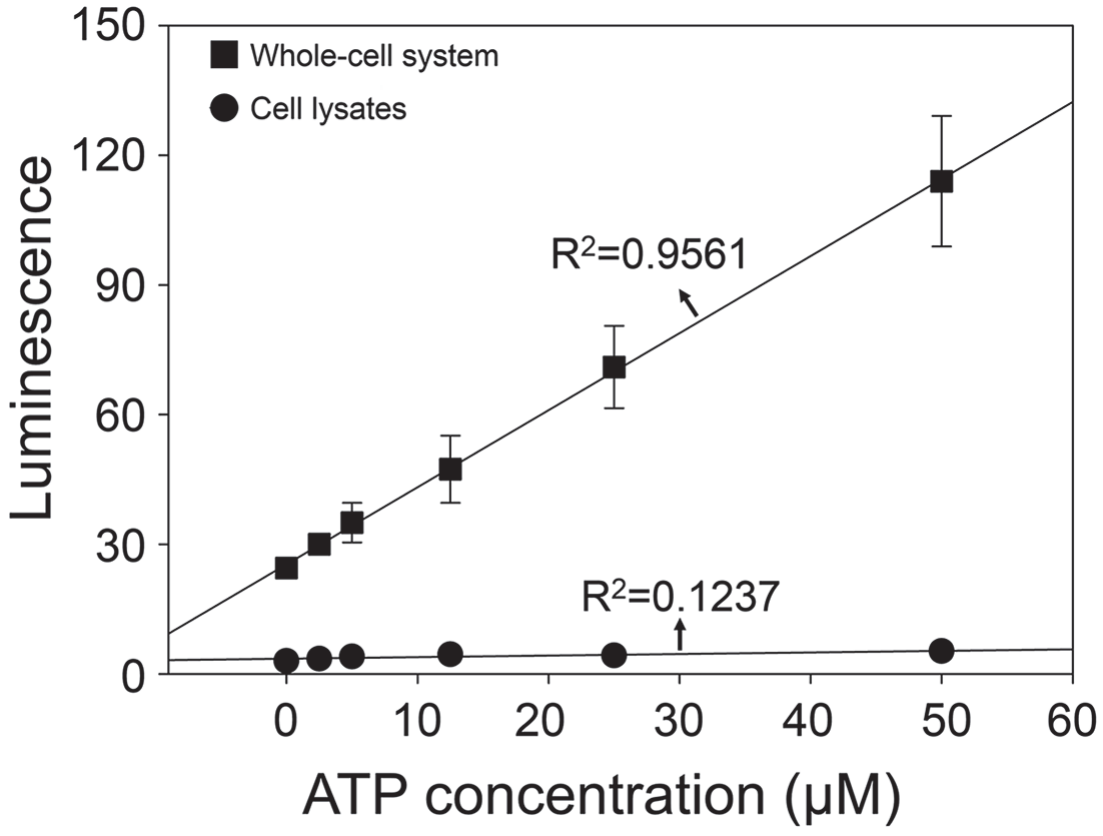
Comparison of linear relationship of bioluminescence intensities generated by the whole-cell system consisting of the engineered *S. boulardii* expressing firefly luciferase or its cell lysates according to the concentration of ATP (0–50 μM) (*n* = 5).

The bioluminescence reaction with FLuc and D-luciferin has been widely used for the detection of ATP because luciferase shows high sensitivity and specificity for ATP [30, 31]. Thus, we evaluated whether the SKCS175 strain expressing FLuc could also detect ATP. As illustrated in Fig. 2, the bioluminescence intensity from the FLuc-expressing SKSC175 strain significantly and proportionally increased with higher ATP concentrations. Moreover, the correlation between ATP concentration and bioluminescence intensity from the SKSC175 strain exhibited a good linear relationship (r^2^ = 0.9561), indicating that this whole-cell-based ATP sensor can be used for the accurate detection and quantification of ATP. However, luminescence signals from the cell lysates of FLuc-expressing SKSC175 strain were not proportionally increased as a function of ATP concentrations, indicative of no linear relationship between luminescence intensities and ATP concentrations (r^2^ = 0.1237). A previous study reported that FLuc-expressing *S. cerevisiae* cells could be used to assess the luciferase activity according to the external ATP concentrations with linearity [32]. However, the sensitivity of our system and *S. cerevisiae*-based whole cell system as ATP sensor cannot be directly compared because there is no information of ATP detection concentration ranges for the *S. cerevisiae*-based whole cell system [32].

### Measurement of Extracellular ATP Levels from DSS-Induced Colitis Mice

**Fig. 3:**
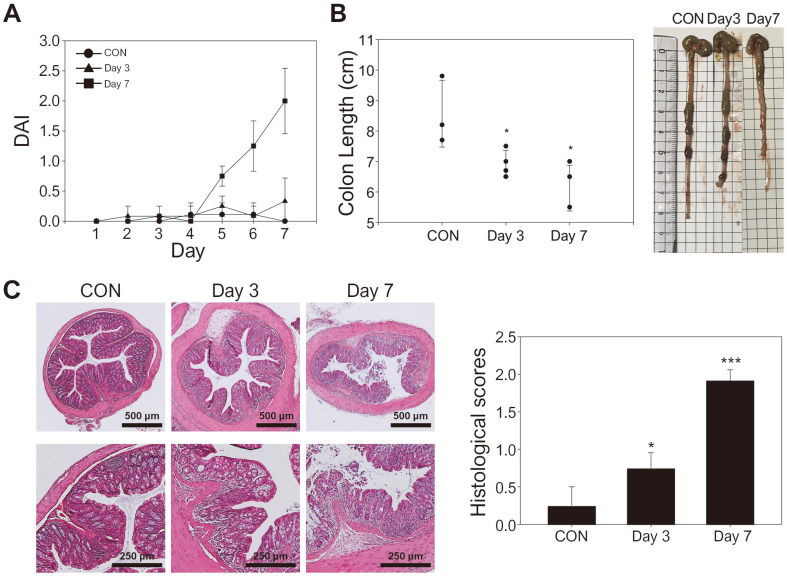
Dextran sulfate sodium (DSS)-induced colitis mouse model assessment. (**A**) Disease activity index (DAI) scores, (**B**) colon lengths, and representative images of each group at different DSS treatment durations. (**C**) H&E-stained sections from the colons at different treatment times and the histological scores. The scale bars represent 1000 and 500 μm for the upper and lower panels, respectively.

We next assessed the ability of our bioluminescence ATP assay system to detect extracellular ATP isolated from colon tissues of a colitis mouse model, as the pathogenesis of colitis is known to be closely associated with high ATP concentrations [22]. Prior to quantifying the ATP from the colon tissues, colitis symptoms were induced by administering the mice with DSS in their drinking water, whereas the healthy group was provided with normal water only. The degree of DSS-induced colitis was then evaluated. As shown in Fig. 3A, the disease activity index (DAI), which is used for evaluating disease status, increased with more prolonged DSS treatment. The mice treated with the DSS solution exhibited shorter colon lengths, which is a representative symptom of IBD (Fig. 3B)[33]. Compared to the control group, the colon length of the mice treated with DSS for 7 days (4.62 ± 0.85 cm) was significantly shorter than that of the control mice (8.57 ± 1.10 cm) and mice treated for 3 days (6.92 ± 0.43 cm). Moreover, histological analysis of the colon tissues via H&E staining demonstrated the occurrence of mucosa damage and the disappearance of crypts in the colon tissue of mice treated with DSS for 7 days, indicating that the disease symptoms became more severe with prolonged DSS treatment (Fig. 3C). These results confirmed that colitis was successfully induced in the mouse model and the pathogenesis of colitis became worse depending on the duration of the DSS treatment.

**Fig. 4:**
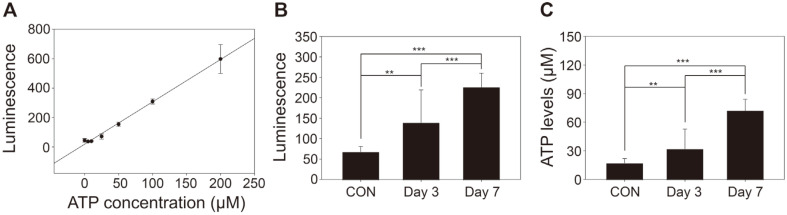
Measurement of extracellular ATP levels in colon of DSS-induced colitis mice using the engineered *S. boulardii* cells. (**A**) Correlation between the bioluminescence intensity and various ATP concentrations (0–200 μM). (**B**) Bioluminescence intensities of the collected supernatants after incubation of the excised colon tissues using the engineered *S. boulardii* expressing firefly luciferase. (**C**) Calculated extracellular ATP levels from colon tissues of DSS-induced colitis mice based on the ATP standard curve (**p < 0.01, ***p < 0.005).

Extracellular ATP levels in colon tissues obtained during the progression of DSS-induced colitis were determined using the whole-cell-based ATP detection assay. An ATP standard curve (0–200 μM) with excellent linearity (R^2^ = 0.9662) was used for the quantification of extracellular ATP levels (Fig. 4A). Luminescence intensities of the collected supernatants after incubation of the excised colon tissues during the progression of colitis were measured using the Fluc-expressing SKSC175 strain and D-luciferin (Fig. 4B), and the ATP levels were then determined (Fig. 4C). Based on the ATP standard curve and bioluminescence intensities, the level of extracellular ATP in the colon tissue of DSS-induced colotis mice tended to increase with disease severity. This result was consistent with the fact that extracellular ATP increased during the progression of colitis due to the release of ATP by the injured/dead cells and activated immune cells [3, 22]. Collectively, our findings demonstrated that the engineered SKSC175 strain has promising potential as a whole-cell-based ATP sensor.

## Conclusion

In this study, we developed a novel ATP bioluminescence assay based on engineered probiotic *Saccharomyces boulardii* expressing firefly luciferase. To achieve this, the gene encoding FLuc was introduced into the genome of *S. boulardii* via the CRISPR/Cas9-based genome editing system. Afterward, the successful expression of FLuc in the intracellular fraction of S .boulardii was confirmed by western blot analysis. Our findings clearly demonstrated that there was a linear relationship between the luminescence intensity generated by the engineered *S. boulardii* and ATP concentration. As a result, the concentrations of ATP in the colon tissues of IBD mice were successfully determined using the proposed whole-cell-based sensor system. Therefore, the whole-cell-based sensor system developed in this study can be applied for the quantitative analysis of the ATP levels, particularly for the diagnosis of ATP-associated diseases such as IBD. In future studies, we intend to investigate the in vivo applications of the whole-cell-based sensor system for ATP detection in diseased models.

## Supplemental Materials

Supplementary data for this paper are available on-line only at http://jmb.or.kr.



## Figures and Tables

**Table 1:** *Saccharomyces boulardii* strains and plasmids used in this study.

Name	Description	Reference
Strains		
*S. boulardii*	ATCC MYA-796	ATCC
SKSC175	*S. boulardii* containing firefly luciferase (FLuc) expression cassette (PGKp-MFα_SP_-FLuc-His_6_-CYC1t)	This study
Plasmids		
pTH652-RLuc/maxFLuc	Source of the gene coding for FLuc	Addgene plasmid #29698
pFLuc	PGKp-MFα_SP_-FLuc-His_6_-CYC1t, 2 μ origin, KanMX^R^	This study
pCas9_AUR	p414-TEF1p-Cas9-CYC1t, modified Cas9 expression plasmid, Aur^R^	[17]
pgRNA-*TRP1*-HYB	SNR52p-*gTRP1*-SUP4t, 2 μ origin, Hyg^R^	[16]
pgRNA-CS6-HYB	SNR52p-*g*CS6-SUP4t, 2 μ origin, Hyg^R^,	This study
