# Supplementary material for: Development of a Novel ATP Bioluminescence Assay Based on Engineered Probiotic Saccharomyces boulardii Expressing Firefly Luciferase
Source: J Microbiol Biotechnol. 2023 Jul 27;33(11):1506–12. doi: 10.4014/jmb.2305.05019 (PMC10699265; doi:10.4014/jmb.2305.05019)
Supplement: Supplementary file 1 [file jmb-33-11-1506-supple.pdf]

J. Microbiol. Biotechnol.
https://doi.org/10.4014/jmb.2305.05019

Supplementary Table and Figure

Table S1. List of primers used in this study.

Name	Sequence (5′→3′)
JC01	ACT AGT ATG AGA TTT CCT TCA ATT TTT ACT GC
JC02	ATT GAA GGA AAT CTC ATA CTA GTC TAG AAT CCG TCG AAA
JC03	GTT ACA AGT CCT CTT CAG AAA TAA GCT TTT GTT CAG CTT CAG CCT CTC TTT TCT
JC04	TTT CTG AAG AGG ACT TGT AAC TCG AGT CAT GTA ATT AGT TAT GTC
JC05	TGA TAT CGA ATT CCT GCA GAG ACG CGA ATT TTT CGA AGA A
JC06	CTG CAG GAA TTC GAT ATC AAG C
JC07	ATT GAA GGA AAT CTC ATA CTA GTT TTT ATA TTT GTT GTA AAA AGT AGA TAA TTA CTT CC
KH13	AAG AAG GGC GGC AAG CAC CAC CAC CAC CAC
KH14	TTC TTA ATG TTC TTA GCG TCT TCA GCT TCA GCC TCT CTT TTC T
KH15	GAA GAC GCT AAG AAC ATT AAG AAG G
KH16	CTT GCC GCC CTT CTT AG
SK33	ATG GAA AAC CGT CAT CAG ATT TAA T

SK34	GAT GTA CAG ATC ATT ATA CGC ACC
SK35	TGA GTT CTT ATA ACC TCG AGG AGA AGT TTT TTT ACC CCT CTC CAC AGA TCA TAT CGA ATT CCT GCA GAG ACG
SK36	GAG AGT AAT TAG GTA GAC CGG GTA GAT TTT TCC GTA ACC TTG GTG TCC CAG TAC CTG GCC GCA AAT TAA
SK37	TCG ATA CTT ATC ATT AAG AAA AGT TTT AGA GCT AGA AAT AGC AAG TTA AAA TAA GG
SK38	AAC TTT TCT TAA TGA TAA GTA TCG ATC ATT TAT CTT TCA CTG CGG A

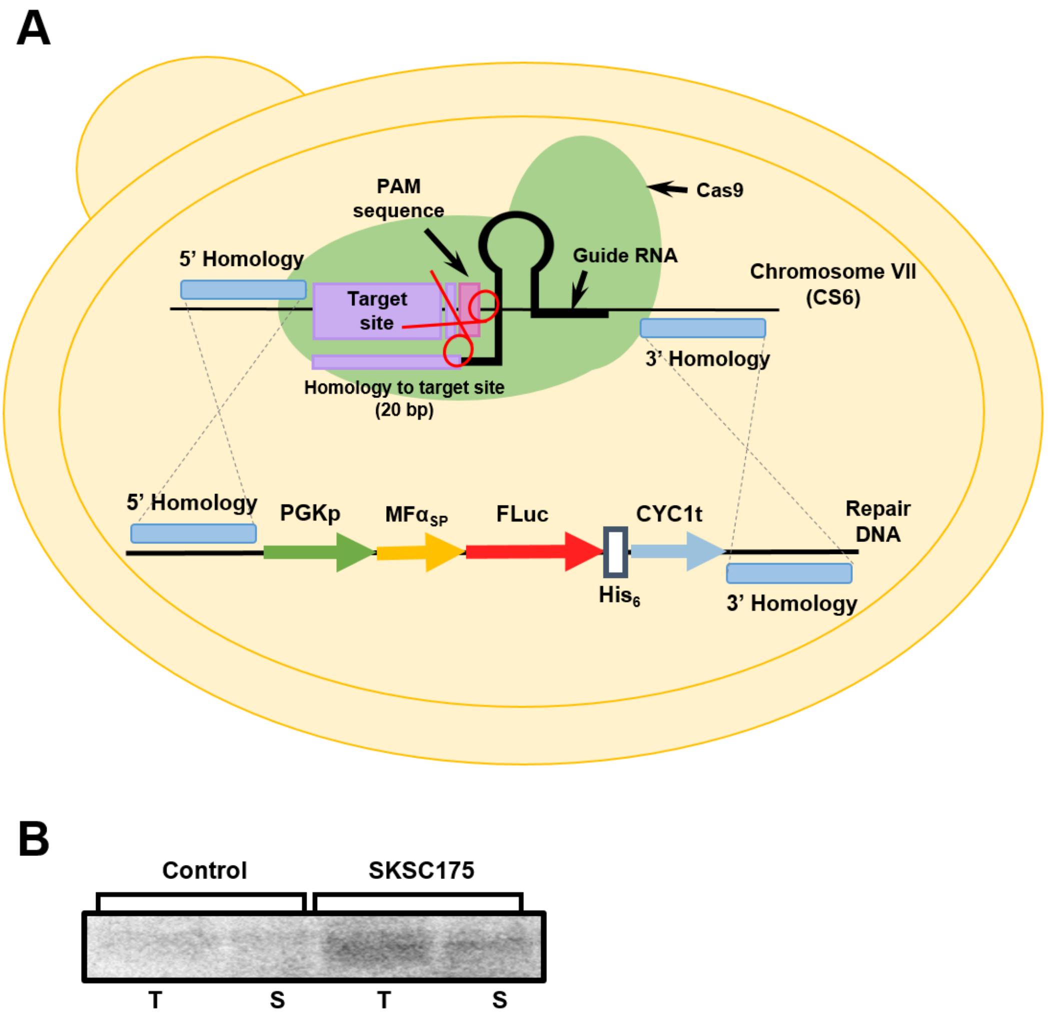

Figure S1. Expression of firefly luciferase in *Saccharomyces boulardii* ATCC MYA-796. (A) Schematic illustration representing the introduction of the fireflily luciferase expression cassette into the genome of *S. boulardii* using the CRISPR/Cas9-based genome editing system. **(B)** Western blot analysis performed to confirm the expression of firefly luciferase. Cell lysates were prepared from wild type *S. boulardii* (control) and the SKSC175 strains and fractionated to obtain the total (T) and soluble (S) fractions. The MFα$_{SP}$-FLuc-His$_6$ protein was detected using an anti-polyhistidine antibody. PAM, protospacer adjacent motif; PGKp, *PGK* promoter; MFα$_{SP}$, mating factor alpha signal peptide; FLuc, firefly luciferase; CYC1t, *CYC1* terminator; SKSC175, *S. boulardii* containing firefly luciferase expression cassette.
